# Detection and monitoring of insect traces in bioaerosols

**DOI:** 10.7717/peerj.10862

**Published:** 2021-02-09

**Authors:** Panyapon Pumkaeo, Junko Takahashi, Hitoshi Iwahashi

**Affiliations:** 1 Division of Science of Biological Resources, United Graduate School of Agricultural Science, Gifu University, Gifu, 1-1 Yanagido, Japan; 2 National Institute of Advanced Industrial Science and Technology (AIST), Health and Medical Research Institute, Tsukuba, Ibaraki, Japan; 3 Department of Applied Life Science, Faculty of Applied Biological Sciences, Gifu University, Gifu, 1-1 Yanagido, Japan

**Keywords:** Particulate matters, Cytochrome-c oxidase I, Operational taxonomic units, Brochosomes

## Abstract

Studies on bioaerosols have primarily focused on their chemical and biological compositions and their impact on public health and the ecosystem. However, most bioaerosol studies have only focused on viruses, bacteria, fungi, and pollen. To assess the diversity and composition of airborne insect material in particulate matter (PM) for the first time, we attempted to detect DNA traces of insect origin in dust samples collected over a two-year period. These samples were systematically collected at one-month intervals and categorized into two groups, PM2.5 and PM10, based on the aerodynamic diameter of the aerosol particles. Cytochrome-c oxidase I (COI) was the barcoding region used to identify the origins of the extracted DNA. The airborne insect community in these samples was analyzed using the Illumina MiSeq platform. The most abundant insect sequences belonged to the order Hemiptera (true bugs), whereas order Diptera were also detected in both PM2.5 and PM10 samples. Additionally, we inferred the presence of particulates of insect origin, such as brochosomes and integument particles, using scanning electron microscopy (SEM). This provided additional confirmation of the molecular results. In this study, we demonstrated the benefits of detection and monitoring of insect information in bioaerosols for understanding the source and composition. Our results suggest that the PM2.5 and PM10 groups are rich in insect diversity. Lastly, the development of databases can improve the identification accuracy of the analytical results.

## Introduction

Bioaerosols are aerosols originating from living organisms and whose particles typically range between 1 nm to up to 10 mm in size (Hinds, 1999). Bioaerosols are composed of pathogenic or non-pathogenic, live or dead bacteria and fungi, viruses, high-molecular-weight allergenic substances, bacterial endotoxins, fungal mycotoxins, pollen, and plant fibers (Douwes et al., 2003; Msa et al., 2005). Bioaerosols can also originate from animals and humans, and include debris from the skin or hair (Després et al., 2012). The impact of aerosol particles on the atmosphere and climate as well as on environmental and human health has resulted in more research being conducted on environmental projects (Šantl-Temkiv et al., 2020; Górny, 2020). Pathogens have been discovered in the atmosphere since the last century, and bioaerosol research has recently been focused on microorganisms (Adams et al., 2013; Nazaroff, 2014; Park et al., 2016; Cox et al., 2020; Kallawicha, Chao & Kotchasatan, 2019; Šantl Temkiv et al., 2020).

The application of molecular techniques in analyzing airborne particles has rapidly increased in the past few years. It has been proven successful in understanding the microbial diversity of indoor and outdoor air samples (Banchi, Pallavicini & Muggia, 2019). Different groups of bioaerosol researchers have performed a wide range of basic and applied scientific studies to gain knowledge on bioaerosols, each within their own objectives and expectations. Several investigations of airborne particles have been conducted to analyze the following: (i) the community composition, abundance, and viability of bacteria, fungi, and plants (Bowers et al., 2009; Adams et al., 2014; Park et al., 2016; Nicolaisen et al., 2017), (ii) airborne allergen-containing particles (Burge & Rogers, 2000; Buters et al., 2015; Kraaijeveld et al., 2015; Bogawski et al., 2016), (iii) airborne pathogenic effects of bioaerosol exposure on human health (Griffin, 2007; Chen & Hildemann, 2009; Fabian et al., 2009; Fronczek & Yoon, 2015; Bhangar et al., 2016; Kobayashi et al., 2016), and (iv) airborne livestock and crop pathogens (Fisher et al., 2012; De Carvalho Ferreira et al., 2013; Brito et al., 2014).

With an estimated 5.5 million species, insects are the most diverse group of animals on the planet (Stork, 2018), and serve as the base of the food web, waste disposal, nutrient cycling, and environmental pollution indicators (Main, 2019). More recently, attention has been given to the declining number and diversity of insects worldwide (Sánchez-Bayo & Wyckhuys, 2019).

However, there is a substantial lack of information on animals, or more specifically, insects from genetic material present in the bioaerosols. Current studies are focusing on addressing the association between bioaerosols and animals (Millner, 2009). A few studies have observed traces of insects in the atmosphere. For example, Wittmaack et al. (2005), by using SEM, observed that bioaerosol samples contain brochosomes and insect scales and do not contain any cells.

Therefore, our study was conducted in order to detect and monitor the traces of insects in bioaerosols. Within this framework, the cytochrome-c oxidase I (COI) region was selected as the barcoding sequence and information was collected from the traces of DNA (owing to the presence of insect particles) in air samples collected over a period of two years. Further, we confirmed the presence of insect integuments using scanning electron microscopy (SEM).

## Materials and Methods

### Aerosol sampling

Aerosol sampling was conducted every month (about 28–30 days) for two years, from 2017 to 2019, on the rooftop of the Gifu Field Science Center, Gifu University, Japan. Eight-stage Andersen AN-200 samplers [aerodynamic diameter (da) = 0.43–0.65, 0.65–1.1, 1.1–2.1, 2.1–3.3, 3.3–4.7, 4.7–7.0, 7.0–1.0, and >11.0 µm; T-Dylec Co., Japan] were used to capture particles on glass fiber that covered a polymer membrane, in order to collect samples from the air. Aerosols were collected at an airflow rate of 20 L min^−1^. The collected samples were classified into two groups. Samples with da >4.7 were included in the large group corresponding to particulate matter 10 (PM10), while those with da <3.3 were placed in the small group corresponding to particulate matter 2.5 (PM2.5).

### DNA extraction and amplification

DNA was extracted from the collected samples using the Extrap Soil DNA Kit Plus ver.2 (Thermo Fisher Scientific, Germany) according to the manufacturer’s protocol. DNA concentration was measured at 260 nm using a micro-volume UV-Vis spectrophotometer Q5000 (Tomy Digital Biology, Tokyo, Japan).

Two-step polymerase chain reaction (PCR) was performed to amplify the COI region. The first PCR amplification was conducted using the universal primers LCO1490 (5′-ggtcaacaaatcataaagatattgg-3′) and HCO2198 (5′-taaacttcagggtgaccaaaaaatca-3′) to amplify a 658-bp fragment of the COI gene (Vrijenhoek, 1994). The reaction mixture contained 12.5 µL of GoTaq^®^ Green Master Mix (2 × solution), 6.1 µL of nuclease-free water, 4 µL of DNA template, and 1.25 µL each of the upstream and downstream primers. The reaction cycle consisted of an initial denaturing step at 94 °C for 3 min, followed by 39 cycles of denaturing at 94 °C for 20 s, annealing at 55 °C for 20 s, and extension at 72 °C for 30 s, with a final extension step at 72 °C for 5 min. Primary PCR products were then purified using the FastGene Gel/PCR Extraction Kit (Nippon Genetics Co. Ltd., Japan).

A second PCR amplification was carried out using primers containing adapter sequences and indexes of 6 nucleotides in length. We used the following primer combination to target a 313 bp COI fragment mlCOIintF (5′-**tcgtcggcagcgtcagatgtgtataagagaca**- gggwacwggwtgaacwgtayccycc-3′) (Leray et al., 2013) with HCO2198 (5′-**gtctcgtgggctcggag- atgtgtataagagacag**xxxxxxtaaacttcagggtgaccaaaaaatca-3′) (Vrijenhoek, 1994) (adapter sequences are bold and index sequences are xxxxxx). For the primer set, we used the optimal reagent concentrations and thermocycler profiles found in the literature (Leray et al., 2013). The GoTaq^®^ Green Master Mix was replaced by the KAPA HiFi HotStart ReadyMix (Roche).

### Sequencing and analysis

Next-generation sequencing was performed by amplicon sequencing on the MiSeq System (Illumina, Inc., USA) at the Gifu University NGS Service Facility.

Raw sequence data of the obtained amplicons underwent demultiplexing, quality-trimming, and quality-filtering with –minqual = 30 (minimum threshold of read quality value) and –maxplowqual = 0.1 (higher rate of lower quality position than minqual), denoising with –primarymaxnmismatch = 0 (the number of mismatches in primary clustering), –secondarymaxnmismatch = 1 (the number of mismatches in secondary clustering), and –pnoisycluster = 0.5 (sensitivity of noise detection) and clustering with –minident = 0.97 (97% of similarity threshold) using the default settings of Claident (https://www.claident.org/), a platform that allows one to complete all steps from sequence processing to molecular identification. This platform uses VSEARCH for quality filtering and assembly procedures. Total operational taxonomic units (OTUs) were clustered with a 97% similarity cutoff using –cducox1 (the Claident Database for UCHIME for animal COX1 (COI) ver.20180412) (Tanabe & Toju, 2013). A search for related species of the organism was performed by BLAST analysis using GenBank (http://www.ncbi.nlm.nih.gov/BLAST/).

The sequences obtained from MiSeq Sequencing have been deposited in the BioProject database under accession numbers PRJNA641822 and PRJNA641864.

### SEM analysis

SEM was performed using the S-4300 SEM instrument (Hitachi, Japan). Chemical fixation is usually applied to biological specimens as a first step in readying them for electron microscopy. Air samples were fixed with osmium tetroxide in order to stabilize them during embedding and to provide resistance to damage during electron beam exposure. The observed bioaerosol particles included both biotic and abiotic components.

## Results

### DNA yield and degree of sequence information

The concentration of DNA extracted from the aerosol samples is shown in Table 1. DNA yield was generally detected in several nanograms per microliter. For example, 1.1 ng µL^−1^ in May 2019 and 1.2 ng µL^−1^ in May 2018. All the samples were generated with less than 20 ng µL^−1^ of DNA. Nevertheless, all the tested samples were successfully amplified using the universal COI primers.

**Table 1:** DNA concentration of Bioaerosols.

Samples	Concentration of DNA (ng/µl)	
	**Small Size**	**Large size**
Aug 2017	–	–
Sep 2017	–	–
Oct 2017	–	5.8
Nov 2017	–	–
Dec 2017	5.1	2.3
Jan 2018	7.2	2.1
Feb 2018	2.0	–
Mar 2018	–	–
April 2018	–	–
May 2018	–	1.2
June 2018	–	–
July 2018	–	–
Aug 2018	3.4	–
Sep 2018	4.0	8.9
Oct 2018	2.1	3.8	
Nov 2018	–	–
Dec2018	3.8	2.5
Jan 2019	–	–
Feb 2019	13.7	4.2
Mar 2019	12.8	6.1
April 2019	1.7	–
May 2019	1.1	10.0
June 2019	12.3	2.3	
July 2019	2.4	–
Aug 2019	7.6	3.1

**Figure 1:**
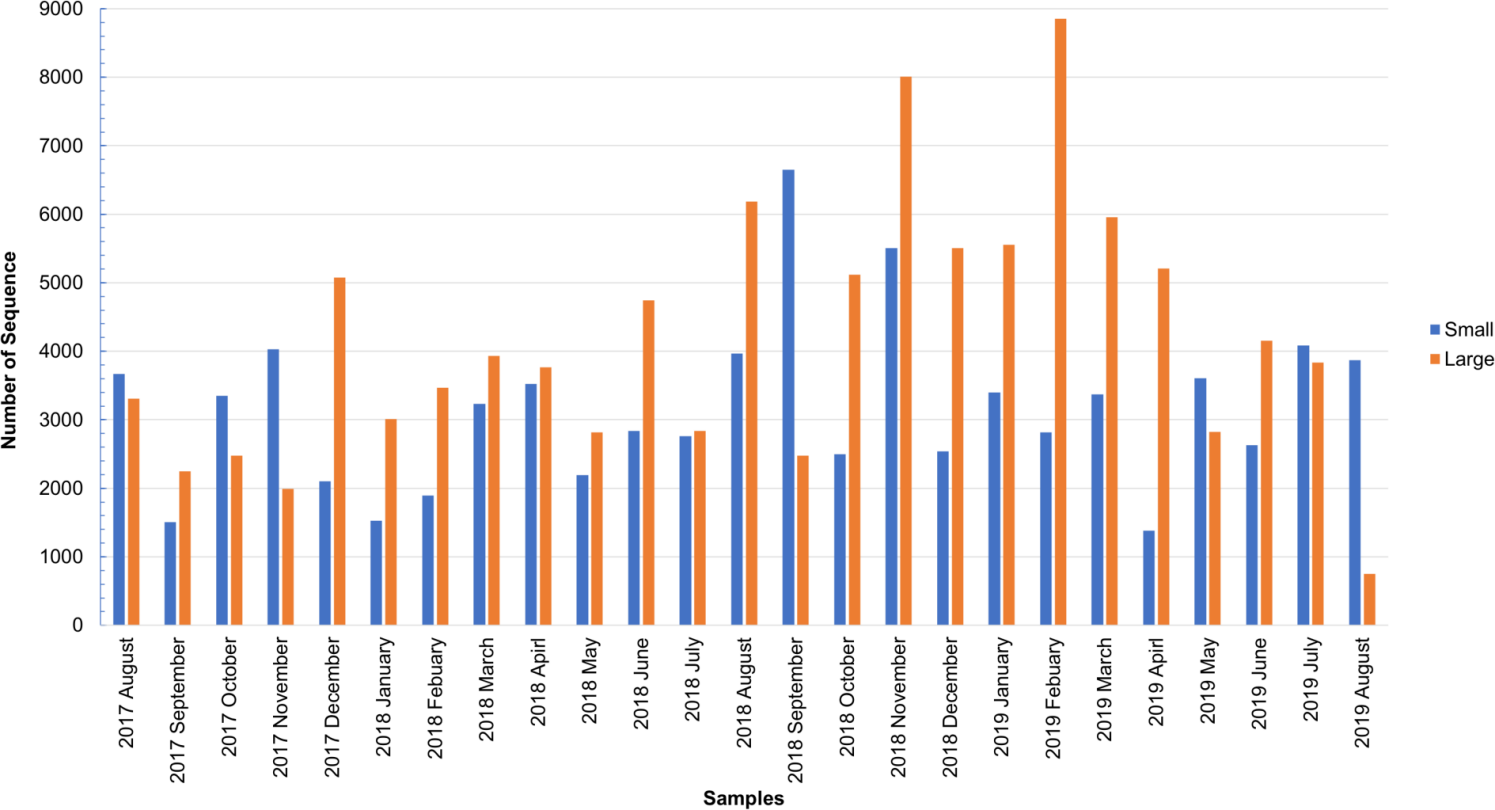
Total number of insect sequences determined by NGS.

As shown in Fig. S1, the total number of sequences obtained from the small and large particle size groups ranged from 6,542 to 29,006 and from 2,798 to 27,431, respectively. The insect sequence was selected from the total number of sequences (Fig. S1), as shown in Fig. 1. The large particle size group was higher than those in the small particle size group. The total insect sequences of the small group were 78,933 (range: 1,378–6,652) and the large group were 104,097 (range: 748–8,853).

**Table 2:** List of insect species identified from bioaerosols.

Accession	Order	Species	NCBI BLAST name	Number of Sequences	
				**Small**	**Large**
MG129828.1	Diptera	*Cecidomyiidae* sp.	flies	2176	2408
GQ864338.1	Sarcoptiformes	*Acarus farris*	mites	85	7
KY270431.1	Araneae	*Allagelena gracilens*	spider	136	438
MF917171.1	Sarcoptiformes	*Anachipteria* sp.	mites	0	7
KJ744479.1	Araneae	*Aname* sp.	spider	0	10
HE587045.1	Hemiptera	*Aphrodes aestuarinus*	bugs	0	11
KR100236.1	Araneae	*Arachnida* sp.	spider	6	0
KX843784.1	Diptera	*Botria subalpina*	flies and midges	8	8
MG988686.1	Hemiptera	*Calinda* sp.	psyllids	0	30
KR569211.1	Hemiptera	*Cedoaphis* sp.	aphids	5	7
KR754495.1	Diptera	*Chironomidae* sp.	flies	58	755
JN301749.1	Diptera	*Chrysotus* sp.	flies	56	0
HF968653.1	Hemiptera	*Cicadellidae* sp.	leafhoppers	0	89
LC329063.1	Diptera	*Cladotanytarsus vanderwulpi*	flies	1	4
MF854444.1	Diptera	*Claustropyga acanthostyla*	flies	0	46
KY433491.1	Diptera	*Culicoides oxystoma*	flies	0	39
KF891942.1	Sarcoptiformes	*Dermatophagoides evansi*	mites & ticks	20	98
MK714193.1	Sarcoptiformes	*Dermatophagoides* sp.	mites & ticks	18	0
JQ555022.1	Lepidoptera	*Epipaschiinae gen. epipaBioLep01* sp.	snout moths	2	22
LC015045.1	Diptera	*Forcipomyia bikanni*	flies	2616	1647
KU496744.1	Diptera	*Geomyza* sp.	flies	613	2324
MF928832.1	Hemiptera	*Hemiptera* sp. BIOUG03439-H02	bugs	170	186
MF928832.1	Hemiptera	*Hemiptera* sp. BIOUG03768-E03	bugs	56230	72665
MK462203.1	Ixodida	*Hyalomma anatolicum*	mites & ticks	87	32
MG357470.1	Lepidoptera	*Hyphantria cunea*	moth	0	8
KR030871.1	Hemiptera	*Hysteroneura setariae*	aphids	129	0
MK015035.1	Trombidiformes	*Isobactrus* sp.	mites & ticks	123	218
JX887510.1	Diptera	*Labrundinia* sp.	flies	0	6
HQ978924.1	Entomobryomorpha	*Lepidosira* sp.	springtails	11	0
KY845478.1	Diptera	*Lispe assimilis*	flies	10	0
AB672744.1	Lithobiomorpha	*Lithobius bicolor*	centipedes	0	6
GU447035.1	Diptera	*Macrocera zetterstedti*	flies	0	6
GU447035.1	Hemiptera	*Mahanarva tristis monagasi*	bugs	0	50
LC319536.1	Haplotaxida	*Megascolecidae* sp. PTP76	annelids	0	5
KX774826.1	Diptera	*Megaselia flavicoxa*	flies	66	265
LC462285.1	Diptera	*Microtendipes famiefeus*	flies	0	14
KR028096.1	Coleoptera	*Mimeoma maculata*	beetles	2803	2885
GU709853.1	Hymenoptera	*Monomorium destructor*	ants	272	301
AF538053.1	Craspedida;	*Monosiga brevicollis*	choanoflagellates	8448	9690
MG104835.1	Diptera	*Mycetophila* sp.	flies	6	0
JX259543.1	Diptera	*Ochlerotatus canadensis*	mosquitos	646	556
LC329188.1	Diptera	*Polypedilum hiroshimaense*	flies	446	3396
HQ928487.1	Diptera	*Psychoda* sp.	flies	1569	3560
KY838040.1	Hymenoptera	*Pteromalidae*	wasps, ants and bees	0	18
KR773901.1	Diptera	*Pteromicra pectorosa*	flies	0	11
JX573674.1	Opiliones	*Sabacon paradoxus*	*daddy long-legs*	0	19
JQ619931.1	Diptera	*Simulium angulistylum*	flies	0	20
KR668236.1	Diptera	*Spelobia bifrons*	flies	0	6
MG823457.1	Diptera	*Symballophthalmus dissimilis*	flies	0	12
KY467177.1	Araneae	*Tetragnatha praedonia*	spiders	2116	2124
KU185080.1	Orthoptera	*Tetrix japonica*	grasshoppers	1	26
MH782433.1	Thysanoptera	*Thrips tabaci*	thrips	0	9
KY307129.1	Hemiptera	*Tinocallis zelkowae*	aphids	0	29
KX496979.1	Entomobryomorpha	*Tomocerus* sp.	springtails	0	19
KY986279.1	Sarcoptiformes	*Tyrophagus longior*	mites & ticks	0	5

### Analysis of insect communities from the sequences

Animal DNA was detected in both the small and large sample groups. Total 299,978 and 348,344 sequences obtained from small and large size samples, respectively, passed quality filtering, and were clustered into 532 OTUs, which showed 315 unique species. Table 2 shows the list of insect species identified from bioaerosols, including 55 species in 16 orders belonging to the phylum Arthropoda. For example, order Hemiptera (leafhopper, true bug), Diptera (fly), Araneae (spider), and Lepidoptera (butterflies).

**Figure 2:**
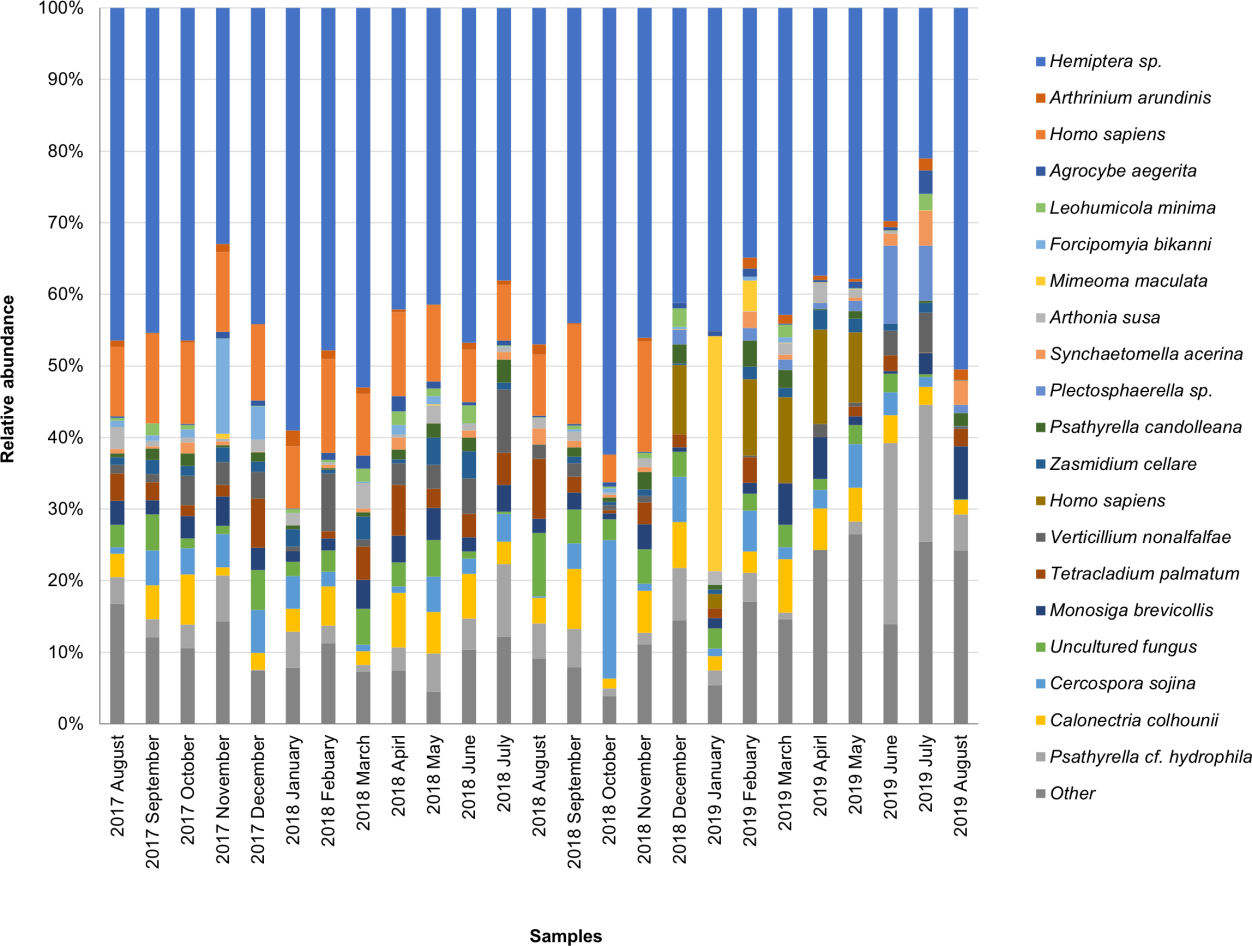
Taxonomic composition of candidate species of outdoor airborne communities (small size samples).

**Figure 3:**
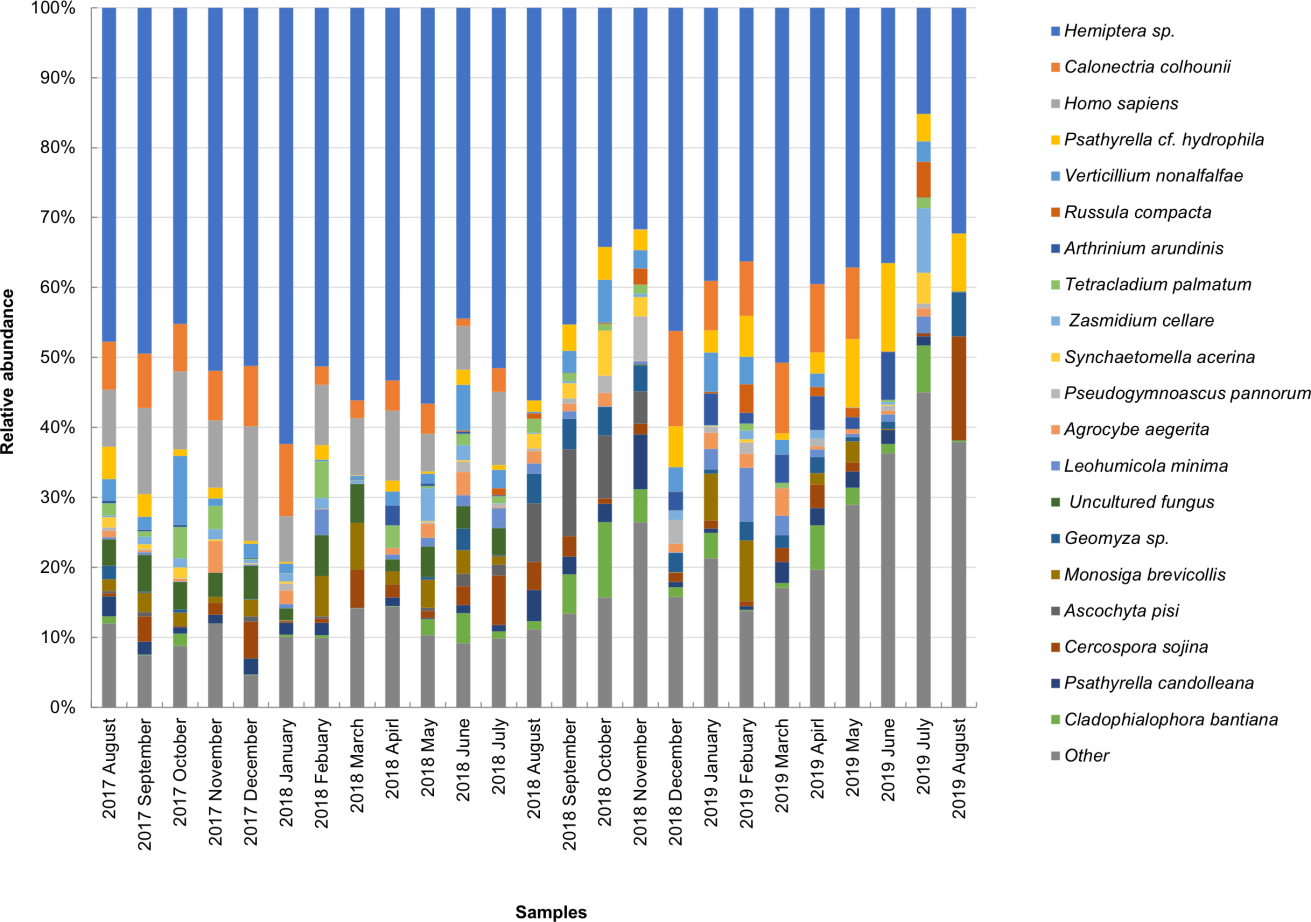
Taxonomic composition of candidate species of outdoor airborne communities (large size samples).

The taxonomic composition of candidate species detected in high abundance from the small and large particle size samples are shown in Tables S1 and S2. The most abundant sequences in both small and large size samples belonged to *Hemiptera* sp., *Homo sapiens*, and *Calonectria colhounii*, while other species corresponded to fewer sequences and only appeared occasionally (Figs. 2 and 3). *Mimeoma maculata* was the insect that was found only in the small size samples and exhibited high abundance in January and February 2019. However, abundant sequences belonging to fungal species, including Ascomycota and Basidiomycota, were obtained as contaminants (Tables S1 and S2). The following taxa, known to contain mainly plant pathogens, were identified: *Calonectria colhounii*, *Plectosphaerella* sp., *Diaporthe longicolla*, *Ascochyta pisi*, and *Cercospora sojina*. *Cladophialophora bantiana* was identified as a human pathogen.

### Evidence confirming the sequence information

Information on airborne insects was only obtained from sequences and may not prove the presence of these insects in the tested samples. To confirm the sequence information, we challenged the confirmation by directly searching for traces of insect material in SEM images. Bacterial, fungal, brochosomal, and other biological particle structures as well as non-biological particles, were clearly visible. The SEM images in Fig. S2 shows the typical morphology of bacterial particles. Figure S3 shows that various fungal particles were present in the bioaerosol samples. Figure S4 shows brochosomes, which are produced by insects. Other biological particles are shown in Fig. S5, and the SEM images in Fig. S6 shows abiotic particles in the aerosol. The brochosomal structures were determined to be an organ of insects from the order Hemiptera, which explained the abundance of OTUs belonging to members of Hemiptera.

## Discussion

Over the past several decades, many researchers have investigated airborne microbial communities in different environments. Our main objective was to monitor and validate the presence of biological traces of animals in aerosols. In this article, we present the results of a two-year study of the airborne insect community at Gifu University, Gifu City, in central Japan, and determined that biological material of insect origin can be found in aerosols (Table 2). The recovery of biological material has reportedly been difficult because of the low biomass concentration. In the present study, DNA was found in both the PM10 and PM2.5 air samples. Although a few samples had DNA concentrations that were too low for detection (Table 1), we successfully amplified DNA from all the tested samples using the universal COI primers and subsequently performed the sequencing reactions.

During winter, the ratio of sequence abundance of small size samples to particle number was low compared to other seasons, in contrast to large size samples. Airborne bioaerosol abundance is lower in winter as it is affected by the temperature (Pietikäinen, Pettersson & Bååth, 2005).

The dominant insect species belonged to the unclassified Hemiptera (order Hemiptera) when BLAST was performed in the GenBank. However, the unclassified Hemiptera is currently labeled as “*Hemiptera* sp. BIOUG03768-E03” by deWaard et al. (2019). Furthermore, the results showed multiple high-scoring hits for numerous fungal taxa. For example, the cause of OTU is closely related to Hemiptera. Searching for OTUs against NCBI Nucleotide using BLAST revealed *Hemiptera* sp., *Cladosporium cladosporioides*, *Cladosporium tenuissimum*, *and Haplothrip stenuipennis*, among which these species become the candidates for high read similarity (data not shown). The multiple high-scoring hits might have occurred from (1) the contaminant sequences that are commonly found in NCBI. Moreover, the continuous increase of novel genome sequences every year leads to an increase in contaminating sequences (Steinegger & Salzberg, 2020). This required improvement and alignment of the database in order to decrease the unnecessary misidentification (2) mismatch of the samples and their index may provide sample identification errors. Accordingly, in one of our investigations on the sample containing formicid DNA, we found that 4% of the sequences (1286 and 1430 of samples 1 and 2, respectively) belonged to other OTUs (Fig. S7). This phenomenon is known as “critical mistagging” (Esling, Lejzerowicz & Pawlowski, 2015). *Hemiptera* sp. was selected as a candidate species in our study as well as other species shown in Figs. 2 and 3 owing to the high score of query covary and high percent identity. In addition, the confirmed presence of Hemiptera in the air sample was investigated. Using specific primers for Hemiptera taxa, the 307 bp fragment in the air samples was successfully amplified (Fig. S8). This finding clearly demonstrates that the air sample contained traces of Hemiptera and confirmed the BLAST against NCBI Nucleotides.

Members of Hemiptera, also known as true bugs, belong to an order of insects that include over 80,000 species. They are a very diverse group comprising insects, aphids, psyllids, as well as Sternorrhyncha (whiteflies), Heteroptera (true bugs), and Auchenorrhyncha (leafhoppers, planthoppers, treehoppers, spittlebugs, and cicadas) (Wilson & Turner, 2010). In Japan, more than 80% of heteropteran species have been identified (Hayashi & Miyamoto, 2005; Ishikawa et al., 2015a). Because of the high habitat diversity and relatively high environmental specificity, the presence, absence, or abundance of different species can act as a bio-indicator of various environmental parameters, such as habitat structure (Ishikawa et al., 2015b).

Other insect species were represented by fewer sequences compared to the dominant ones. Sequences of *M. maculata* were found only in the samples collected in January and February 2019, and were not detected in the samples from 2018, and this study only recorded a single replicate. Thus, we were unable to ascertain whether the occurrence of this species was season specific. We found several sequences belonging to order Araneae (spider) in the air samples. For example, *A. gracilens, Aname* sp., *Arachnida* sp., and *Tetragnatha praedonia* (Table 2). Spiders do not usually come to mind when we describe airborne organisms. However, these wingless arthropods have been found 4 km up in the air and disperse hundreds of kilometers by ballooning (Glick, 1939; Després et al., 2012; Guarino, 2018; Morley & Robert, 2018). Ballooning is the behavior of some spiders who use air dispersal to move from one location to another (Sheldon et al., 2017). *Chironomidae* sp., which are non-biting midges that act as important indicator organisms, were also found in this study (Table 2). The distribution of this species in freshwater bodies indicates the presence of pollution in the environment, and is useful for the study of past environmental conditions in affected regions (Francis, 2004; MacDonald et al., 2009; McKeown & Potito, 2016). The relationship between midges and environmental factors can be applied to further our understanding of past environmental changes.

Species belonging to Cecidomyiidae under the order Diptera were one of the insect species presented in this study (Table 2). The Asian Rice Gall Midge (*Orseolia oryzae*) is a member of the Cecidomyiidae family, and is a major pest of rice that causes severe damage leading to yield losses in India, Thailand, and several other Asian countries (Bentur et al., 2004; Atray, Bentur & Nair, 2015; Janique et al., 2017). Although there are no reports on this crop pest in Japan, according to the long-range transport of aerosols, some studies have shown that aerosols produced in Asia can be transported to locations as far as North America (Lin et al., 2012; Cooper et al., 2015; Verstraeten et al., 2015; Martins et al., 2018). However, some studies suggest the possibility that microorganisms can travel long distances, such as from China to Japan (Wu et al., 2004; Hua et al., 2007; Kakikawa et al., 2008; Maki et al., 2008; Iwasaka et al., 2009). Our results suggest that the high abundance of this organism in the tested environment indicates the potential of applying these analytical tools to detect the biological interactions among important pests in specific locations.

However, this barcode region has been observed in fungal species and is an effective recognition tool in a number of studies (Seifert et al., 2007; Dentinger, Didukh & Moncalvo, 2011). Thus, majority of the aerosol samples were dominated by *C. colhounii* in small and large samples (Tables S1 and S2). These belong to the division Ascomycota, known as the division with the most airborne fungi (including Basidiomycota) frequently found in the atmosphere (Fröhlich-Nowoisky et al., 2009). Moreover, these results might be attributed to the fact that fungi are the most common organisms found in aerosols (Elbert et al., 2007; Bauer et al., 2008; Crawford et al., 2009; Després et al., 2012). In addition, although it is generally known that majority of the fungal genetic material in aerosols is acquired from fungal spores, fungal DNA may also be derived from other fungal materials such as hyphae and tissue fragments (Després et al., 2012).

SEM image monitoring was employed in the present study to confirm the sequence information. The SEM images revealed that the majority of airborne particles commonly found in the atmosphere included both biotic and abiotic particles. Airborne microbes are a common group of bioaerosols and are detailed in the review by Després et al. (2012). The aerosol particles shown in Fig. S2 were most likely bacterial cells that presented rod and coccus-like shapes (Tang et al., 2018). Based on the classification of fungal particles (Wittmaack et al., 2005; Valsan et al., 2015), the particles shown in Fig. S3 were considered as conidia and spores, and were most likely spores of fungi from the taxa Ascomycota. This determination concurred with the results of our sequence analysis.

In the present study, the brochosomes detected by SEM resulted from the emission of solid secretions from insects that frequently appeared in bioaerosols, as reported in a number of studies (Wittmaack et al., 2005; Kang et al., 2012; Li et al., 2019). Brochosomes are 250–300 nm in diameter and are morphologically very similar to a football (Fig. S4) (also known as a soccer ball in the USA), and resemble C60 Buckminster Fullerenes (Wittmaack et al., 2005). They are comprised mainly of proteins (60%–70%) (Rakitov et al., 2018). Brochosomes are produced intracellularly in specialized glandular segments of the Malpighian tubules of insects, especially of leafhoppers (family Cicadellidae in the order Hemiptera), and have been investigated to serve as a very efficient water-repellent protective surface coating (Wittmaack, 2005; Rakitov, 2009). Consequently, our results clearly demonstrated that the highest abundance of *Hemiptera* sp. (Figs. 2 and 3) came from the brochosomes, which are organs of insects from the order Hemiptera (Rakitov, 2002; Wyniger et al., 2008). It should be noted that the brochosomes we observed were agglomerated and accompanied with other flakes. These flakes may contain DNA fragments from their original organisms.

## Conclusion

The present study demonstrated that the short primer target of the 313-bp insect COI fragment could be detected in bioaerosols using the next-generation sequencing platform. The environmental factors (seasonal) did not affect the sequence abundance and taxonomic composition of the community, as observed when the small and large size samples were compared. The dominant insect species found in the air samples was Hemiptera sp. The diversity of fungi, which mostly belonged to the groups Ascomycota and Basidiomycota, was greater than that of insect species, due to high emission of fungal spores into the environment. In addition, the observation of the aerosol samples by SEM determined that fungal spores and brochosomes were often of biological origin, thereby confirming the sequence information.

Our work suggests that bioaerosol monitoring using next-generation sequencing can provide useful information regarding the concentrations of various aerobiological constituents. The results obtained during the collection were generally consistent with the SEM observations. However, the validation of these aspects necessitates future studies. In particular, our biological information is largely based on the databases, which are still developing. Thus, it should be noted that the development of databases, such as the detection method of contaminating sequences, can improve the identification accuracy of the analytical results.

##  Supplemental Information

10.7717/peerj.10862/supp-1 Supplemental Information 1 Summary of taxonomic information of high abundance detection on small size samples

10.7717/peerj.10862/supp-2 Supplemental Information 2 Summary of taxonomic information of high abundance detection on large size samples

10.7717/peerj.10862/supp-3 Supplemental Information 3 Total number of sequences determined by NGS.

10.7717/peerj.10862/supp-4 Supplemental Information 4 SEM images of bacterial cells. (a) rod-shaped bacterial cell, aggregated; (b) rod-shaped bacterial cell; (c) coccus-shaped bacterial cells; (d) coccus- and short rod-shaped bacterial cells.

10.7717/peerj.10862/supp-5 Supplemental Information 5 SEM images of various types of fungal spores.

10.7717/peerj.10862/supp-6 Supplemental Information 6 SEM images of the brochosomes. (a–b) group of brochosomes; (c–d) brochosomes at high magnification.

10.7717/peerj.10862/supp-7 Supplemental Information 7 SEM images of the other biological particles. (a) vegetal debris; (b–c) plant fragments; (d) unclassified diatom.

10.7717/peerj.10862/supp-8 Supplemental Information 8 SEM images of non-biological particles. (a) complex structure of dust; (b–d) fly ash; (e) crystal-shaped particles; (f) sodium chloride particles; (g–h) aggregated soot; (i) soot aggregated with fly ash.

10.7717/peerj.10862/supp-9 Supplemental Information 9 Taxonomic composition from formicid specimen.

10.7717/peerj.10862/supp-10 Supplemental Information 10 Detection of Hemiptera derived sequences from Hemiptera and aerosol samples (a) Amplification of COI region by specific primers. lane1(Insect No.1), lane2 (Insect No.2), lane3 (Insect No.3) lane4 (Insect No.4), lane5 (aerosol sample), lane6 (aerosol sample), laneM (DNA maker) (b) Insect samples in the order Hemiptera Primer: LepF2_t1 5′-TGTAAAACGACGGCCAGTAATCATAARGATATYGG-3′MHemR 5′-GGTGGATAAACTGTTCAWCC-3′Reference: https://doi.org/10.1139/gen-2018-0093.

## Additional Information and Declarations

### DNA Deposition

The following information was supplied regarding the deposition of DNA sequences: Sequences are available at NCBI: PRJNA641822 and PRJNA641864.

### Data Availability

The following information was supplied regarding data availability: Data are available as Supplementary Files.
