# Supplementary material for: Detection and monitoring of insect traces in bioaerosols
Source: PeerJ. 2021 Feb 9;9:e10862. doi: 10.7717/peerj.10862 (PMC7879950; doi:10.7717/peerj.10862)
Supplement: Supplemental Information 1 [file peerj-09-10862-s001.docx]

**Table.S1** Summary of taxonomic information of high abundance detection on small size samples.

| Kingdom | Phylum | Class | Oder | Family | Genus | Species | Accession | Number of sequences |
| --- | --- | --- | --- | --- | --- | --- | --- | --- |
| Metazoa | Arthropoda | Insecta | Hemiptera |  |  | unclassified Hemiptera | MF928832.1 | 130768 |
| Metazoa | Chordata | Mammalia | Primates | Hominidae | Homo | *Homo sapiens* | MH973718.1 | 21359 |
| Fungi | Basidiomycota | Agaricomycetes | Agaricales | Psathyrellaceae | Psathyrella | *Psathyrella cf. hydrophila* | JN029501.1 | 16629 |
| Fungi | Ascomycota | Sordariomycetes | Hypocreales | Nectriaceae | Calonectria | *Calonectria colhounii* | JN574872.1 | 13454 |
| Fungi | Ascomycota | Dothideomycetes | Mycosphaerellales | Mycosphaerellaceae | Cercospora | *Cercospora sojina* | KC888822.1 | 12398 |
|  |  | Choanoflagellata | Craspedida | Salpingoecidae | Monosiga | *Monosiga brevicollis* | AF538053.1 | 8448 |
| Fungi | Ascomycota |  |  |  | Tetracladium | *Tetracladium palmatum* | EU883403.1 | 7704 |
| Fungi | Ascomycota | Sordariomycetes | Glomerellales | Plectosphaerellaceae | Verticillium | *Verticillium nonalfalfae* | KR704425.1 | 7282 |
| Fungi | Ascomycota | Dothideomycetes | Mycosphaerellales | Mycosphaerellaceae | Zasmidium | *Zasmidium cellare* | NC_030334.1 | 3880 |
| Fungi | Basidiomycota | Agaricomycetes | Agaricales | Psathyrellaceae | Psathyrella | *Psathyrella candolleana* | JN029500.1 | 3854 |
| Fungi | Ascomycota | Sordariomycetes | Glomerellales | Plectosphaerellaceae | Plectosphaerella | *Plectosphaerella* sp. | MK697668.1 | 3811 |
| Fungi | Ascomycota | Leotiomycetes | Helotiales | Chaetomellaceae | Synchaetomella | *Synchaetomella acerina* | JX989833.1 | 3496 |
| Fungi | Ascomycota | Arthoniomycetes | Arthoniales | Arthoniaceae | Arthonia | *Arthonia susa* | MH015348.1 | 3206 |
| Metazoa | Arthropoda | Insecta | Coleoptera | Scarabaeidae | Mimeoma | *Mimeoma maculata* | KR028096.1 | 2803 |
| Metazoa | Arthropoda | Insecta | Diptera | Ceratopogonidae | Forcipomyia | *Forcipomyia bikanni* | LC015045.1 | 2616 |
| Fungi | Ascomycota | Leotiomycetes |  |  | Leohumicola | *Leohumicola minima* | EU678466.1 | 2352 |
| Fungi | Basidiomycota | Agaricomycetes | Agaricales | Bolbitiaceae | Agrocybe | *Agrocybe aegerita* | MF979820.1 | 2282 |
| Fungi | Ascomycota | Sordariomycetes | Xylariales | Apiosporaceae | Arthrinium | *Arthrinium arundinis* | KY775582.1 | 2181 |
