# Supplementary material for: Detection and monitoring of insect traces in bioaerosols
Source: PeerJ. 2021 Feb 9;9:e10862. doi: 10.7717/peerj.10862 (PMC7879950; doi:10.7717/peerj.10862)
Supplement: Supplemental Information 2 [file peerj-09-10862-s002.docx]

**Table.S2** Summary of taxonomic information of high abundance detection in large size samples.

| Kingdom | Phylum | Class | Oder | Family | Genus | Species | Accession | Number of sequences |
| --- | --- | --- | --- | --- | --- | --- | --- | --- |
| Metazoa | Arthropoda | Insecta | Hemiptera |  |  | unclassified Hemiptera | MF928832.1 | 152657 |
| Fungi | Ascomycota | Sordariomycetes | Hypocreales | Nectriaceae | Calonectria | *Calonectria colhounii* | JN574872.1 | 17104 |
| Metazoa | Chordata | Mammalia | Primates | Hominidae | Homo | *Homo sapiens* | MH973718.1 | 13137 |
| Fungi | Basidiomycota | Agaricomycetes | Agaricales | Psathyrellaceae | Psathyrella | *Psathyrella cf. hydrophila* | JN029501.1 | 11163 |
| Fungi | Ascomycota | Sordariomycetes | Glomerellales | Plectosphaerellaceae | Verticillium | *Verticillium nonalfalfae* | KR704425.1 | 9812 |
| Fungi | Ascomycota | Eurotiomycetes | Chaetothyriales | Herpotrichiellaceae | Cladophialophora | *Cladophialophora bantiana* | KX257489.1 | 9419 |
| Fungi | Basidiomycota | Agaricomycetes | Agaricales | Psathyrellaceae | Psathyrella | *Psathyrella candolleana* | JN029500.1 | 7825 |
| Fungi | Ascomycota | Dothideomycetes | Mycosphaerellales | Mycosphaerellaceae | Cercospora | *Cercospora sojina* | KC888822.1 | 7393 |
| Fungi | Ascomycota | Dothideomycetes | Pleosporales | Didymellaceae | Ascochyta | *Ascochyta pisi* | MK468491.1 | 7316 |
|  |  | Choanoflagellata | Craspedida | Salpingoecidae | Monosiga | *Monosiga brevicollis* | AF538053.1 | 7160 |
| Metazoa | Arthropoda | Insecta | Diptera | Opomyzidae | Geomyza | *Geomyza* sp. | KU496744.1 | 6441 |
| Fungi | Ascomycota | Leotiomycetes |  |  | Leohumicola | *Leohumicola minima* | EU678466.1 | 5273 |
| Fungi | Basidiomycota | Agaricomycetes | Agaricales | Bolbitiaceae | Agrocybe | *Agrocybe aegerita* | MF979820.1 | 4705 |
| Fungi | Ascomycota | Leotiomycetes |  | Pseudeurotiaceae | Pseudogymnoascus | *Pseudogymnoascus pannorum* | KR055655.1 | 4502 |
| Fungi | Ascomycota | Leotiomycetes | Helotiales | Chaetomellaceae | Synchaetomella | *Synchaetomella acerina* | JX989833.1 | 4480 |
| Fungi | Ascomycota | Dothideomycetes | Mycosphaerellales | Mycosphaerellaceae | Zasmidium | *Zasmidium cellare* | NC_030334.1 | 4274 |
| Fungi | Ascomycota |  |  |  | Tetracladium | *Tetracladium palmatum* | EU883403.1 | 4186 |
| Fungi | Ascomycota | Sordariomycetes | Xylariales | Apiosporaceae | Arthrinium | *Arthrinium arundinis* | KY775582.1 | 4171 |
| Fungi | Basidiomycota | Agaricomycetes | Russulales | Russulaceae | Russula | *Russula compacta* | NC_037773.1 | 3205 |
