# Supplementary figures and images for: Detection and monitoring of insect traces in bioaerosols
Source: PeerJ. 2021 Feb 9;9:e10862. doi: 10.7717/peerj.10862 (PMC7879950; doi:10.7717/peerj.10862)

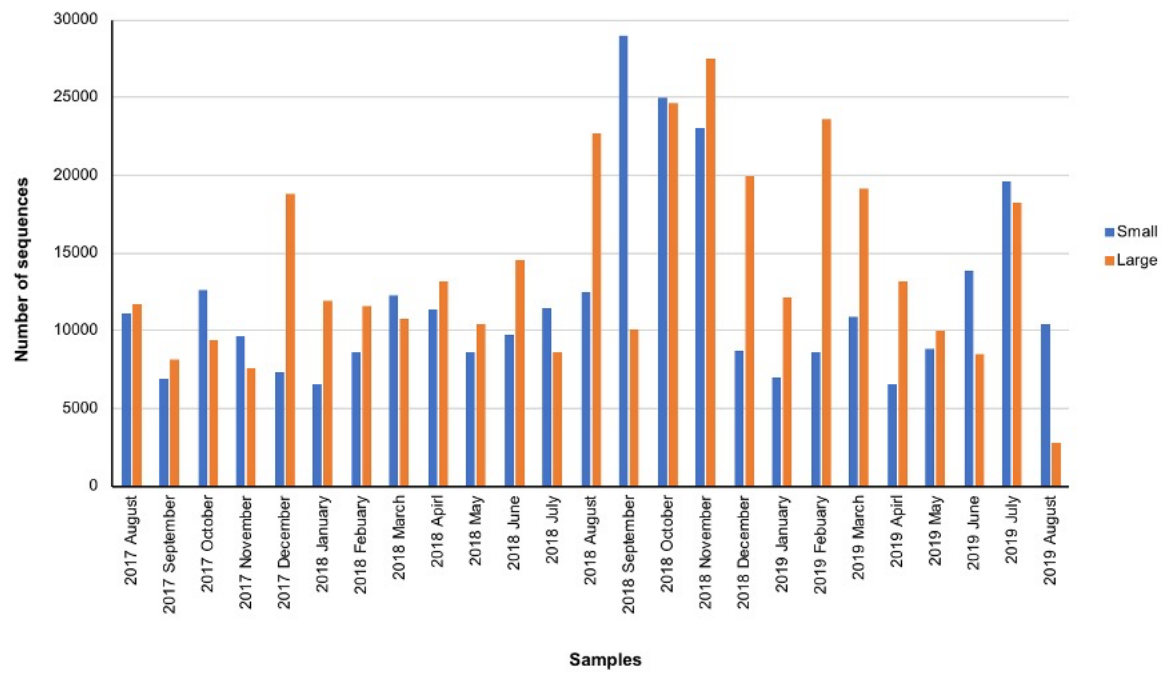

Supplement: Supplemental Information 3 [file peerj-09-10862-s003.pdf]

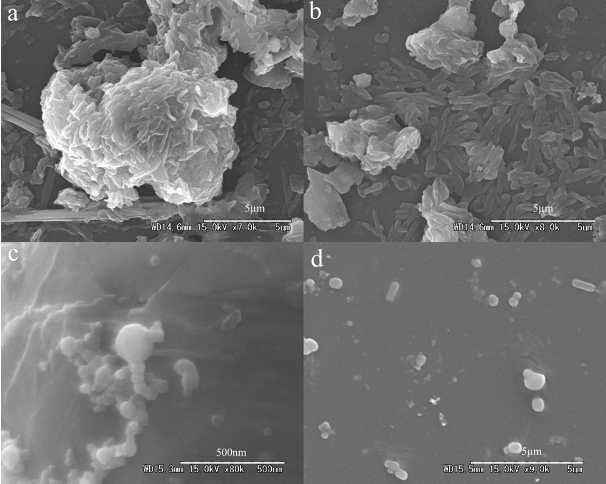

Supplement: Supplemental Information 4 — (a) rod-shaped bacterial cell, aggregated; (b) rod-shaped bacterial cell; (c) coccus-shaped bacterial cells; (d) coccus- and short rod-shaped bacterial cells. [file peerj-09-10862-s004.png]

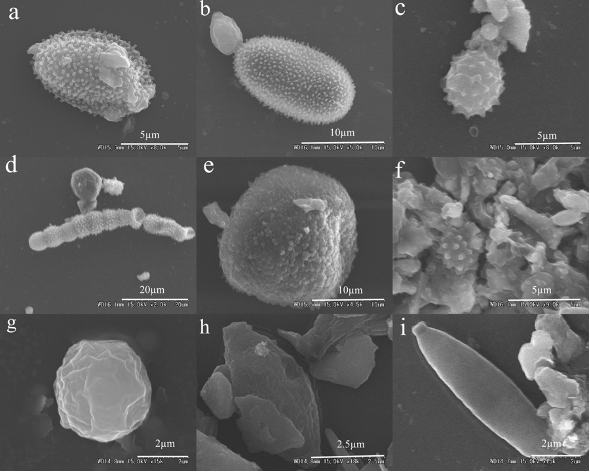

Supplement: Supplemental Information 5 [file peerj-09-10862-s005.png]

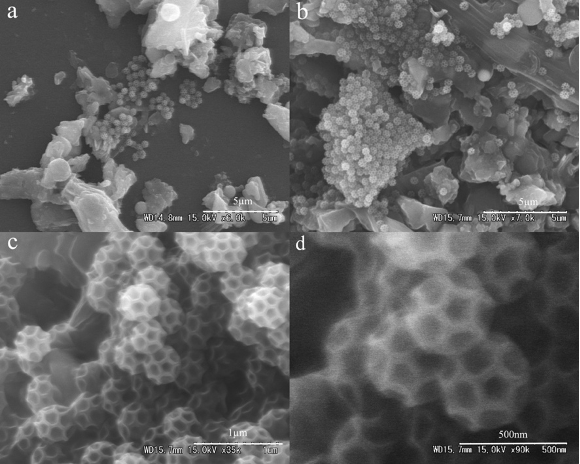

Supplement: Supplemental Information 6 — (a–b) group of brochosomes; (c–d) brochosomes at high magnification. [file peerj-09-10862-s006.png]

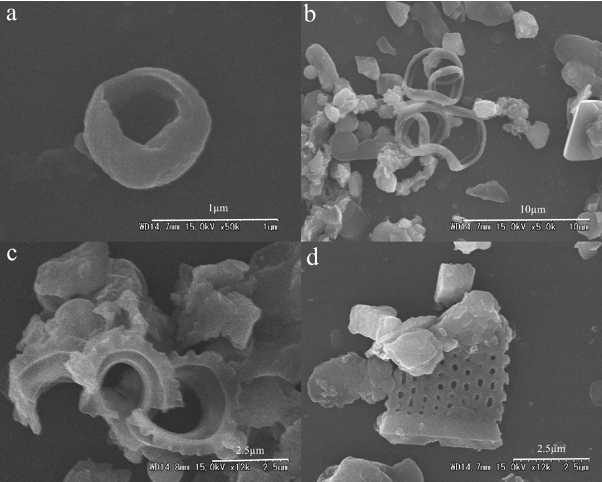

Supplement: Supplemental Information 7 — (a) vegetal debris; (b–c) plant fragments; (d) unclassified diatom. [file peerj-09-10862-s007.png]

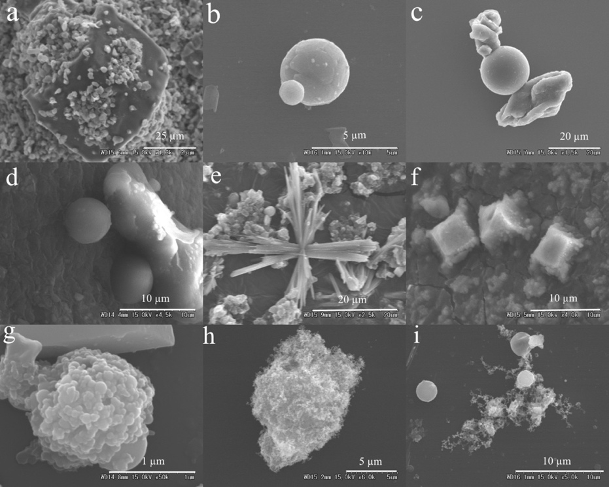

Supplement: Supplemental Information 8 — (a) complex structure of dust; (b–d) fly ash; (e) crystal-shaped particles; (f) sodium chloride particles; (g–h) aggregated soot; (i) soot aggregated with fly ash. [file peerj-09-10862-s008.png]

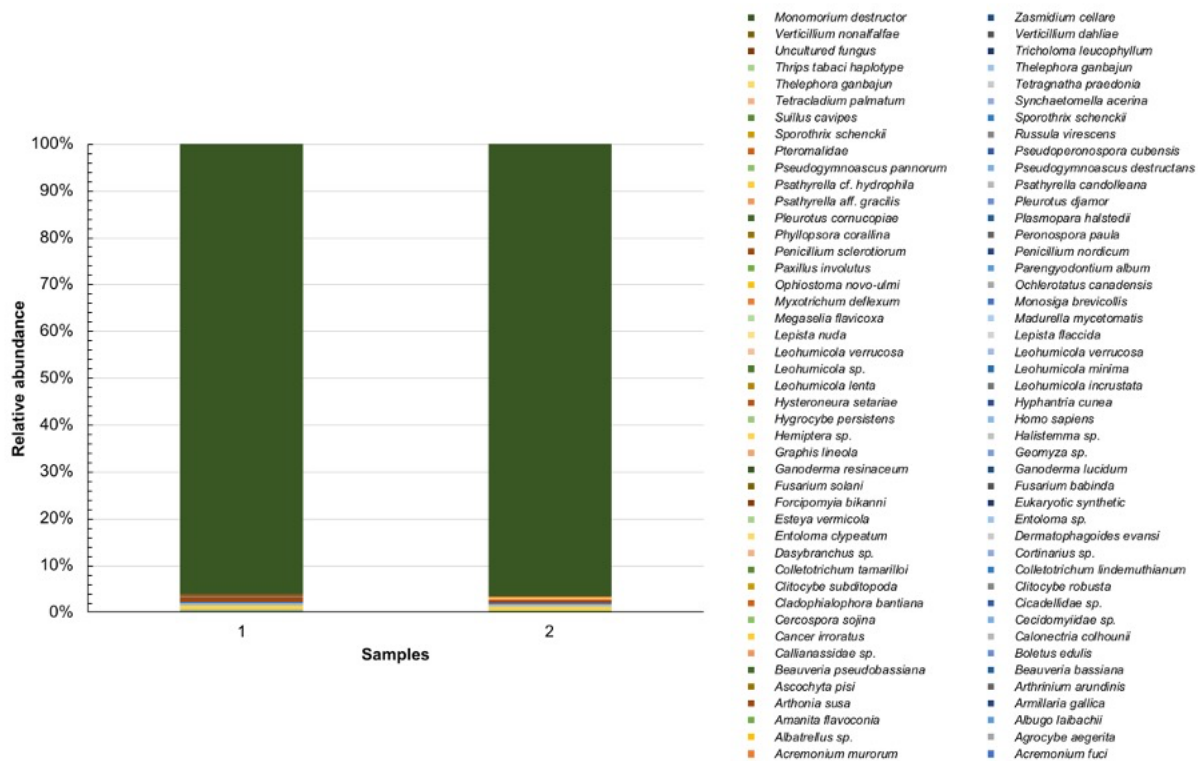

Supplement: Supplemental Information 9 [file peerj-09-10862-s009.pdf]

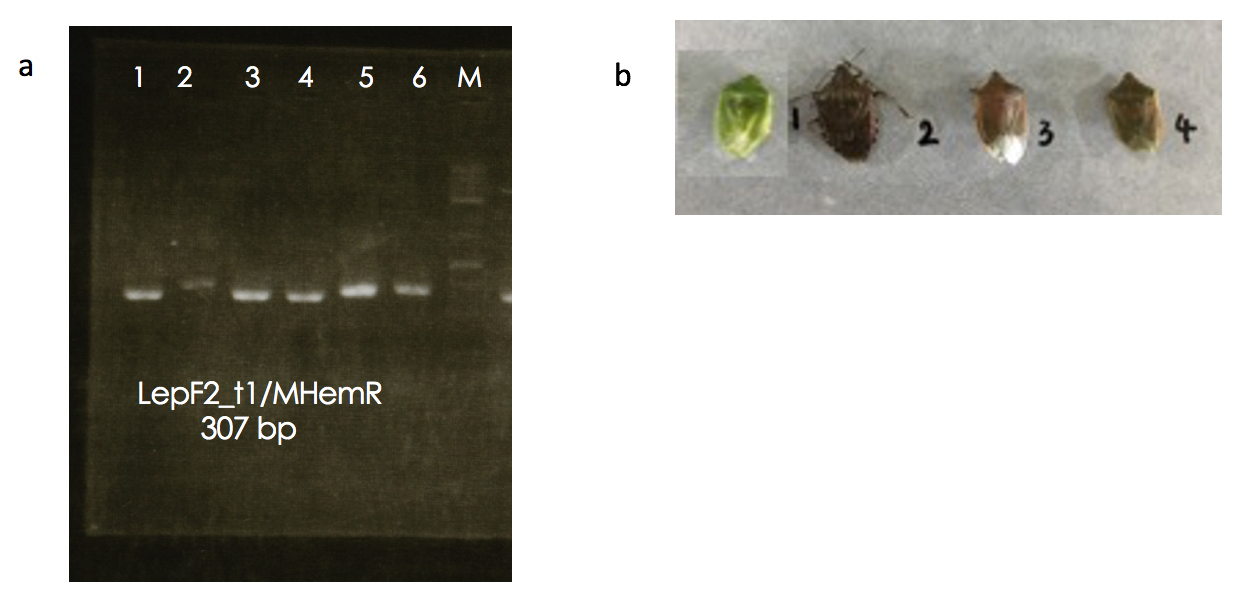

Supplement: Supplemental Information 10 — (a) Amplification of COI region by specific primers. lane1(Insect No.1), lane2 (Insect No.2), lane3 (Insect No.3) lane4 (Insect No.4), lane5 (aerosol sample), lane6 (aerosol sample), laneM (DNA maker) (b) Insect samples in the order Hemiptera Primer: LepF2_t1 5′-TGTAAAACGACGGCCAGTAATCATAARGATATYGG-3′MHemR 5′-GGTGGATAAACTGTTCAWCC-3′Reference: https://doi.org/10.1139/gen-2018-0093. [file peerj-09-10862-s010.png]
